# Can Frailty Be a Predictor of ICD Shock after the Implantation of a Cardioverter Defibrillator in Elderly Patients?

**DOI:** 10.3390/s21186299

**Published:** 2021-09-20

**Authors:** Agnieszka Mlynarska, Rafal Mlynarski, Bartosz Uchmanowicz, Wioletta Mikuľáková

**Affiliations:** 1 Department of Gerontology and Geriatric Nursing, School of Health Sciences, Medical University of Silesia, 40-635 Katowice, Poland; 2 Upper Silesian Heart Centre, Department of Electrocardiology, 40-055 Katowice, Poland; rmlynarski@sum.edu.pl; 3 Department of Electrocardiology and Heart Failure, School of Health Sciences, Medical University of Silesia, 40-635 Katowice, Poland; 4 Department of Clinical Nursing, Faculty of Health Sciences, Wroclaw Medical University, 51-618 Wroclaw, Poland; bartosz.uchmanowicz@umed.wroc.pl; 5 Department of Physiotherapy, Faculty of Health Care, University of Presov, Partizánska 1, 08001 Presov, Slovakia; wioletta.mikulakova@unipo.sk

**Keywords:** frailty, implantable cardioverter-defibrillator, electric shock, inadequate electric shocks

## Abstract

Introduction: The aim of the study was to assess the prevalence of frailty among elderly patients who had an implanted cardioverter defibrillator, as well as the influence of frailty on the main endpoints during the follow-up. Methods: The study included 103 patients > 60 years of age (85M, aged 71.56–8.17 years). All of the patients had an implanted single or dual-chamber cardioverter-defibrillator. In the research, there was a 12-month follow-up. The occurrence of frailty syndrome was assessed using the Tilburg Frailty Indicator scale (TFI). Results: Frailty syndrome was diagnosed in 75.73% of the patients that were included in the study. The mean values of the TFI were 6.55 ± 2.67, in the physical domain 4.06 ± 1.79, in the psychological domain 2.06 ± 1.10, and in the social domain 0.44 ± 0.55. During the follow-up period, 27.2% of patients had a defibrillator cardioverter electric shock, which occurred statistically more often in patients with diagnosed frailty syndrome (34.6%) compared to the robust patients (4%); *p* = 0.0062. In the logistic regression, frailty (OR: 1.203, 95% CI:1.0126–1.4298; *p* < 0.030) was an independent predictor of a defibrillator cardioverter electric shock. Similarly, in the logistic regression, frailty (OR: 1.3623, 95% CI:1.0290–1.8035; *p* = 0.019) was also an independent predictor for inadequate electric shocks. Conclusion: About three-quarters of the elderly patients that had qualified for ICD implantation were affected by frailty syndrome. In the frailty subgroup, adequate and inadequate shocks occurred more often compared to the robust patients.

## 1. Introduction

Implanting a cardioverter-defibrillator (ICD) is a recognized method for preventing sudden cardiac death due to ventricular tachyarrhythmias. The main purpose of ICD implantation is to stop arrhythmias. This function is realized in two ways: by conducting the so-called antiarrhythmic pacing (ATP—anti-tachycardia pacing) or by causing a device electric shock (shock). When ventricular fibrillation (VF) occurs, the defibrillator delivers an electric shock. The electric shock has 30–40 J energy, which enables almost 100% of VF episodes to be interrupted; however, it may be characterized by severe pain. How traumatic this experience can be for a patient is illustrated by the results of studies that indicate that about 5% of patients who have experienced numerous ICD shocks would not agree to have a device implanted again [1,2,3]. One of the biggest problems in patients with an implanted ICD is unjustified device interventions, or electrical storms, which can affect up to 15–20% of patients. Supraventricular tachyarrhythmias are the most common cause, followed by incorrect arrhythmia counting or lead damage. About 70% of those who are treated as the primary prevention and about 45–50% as the secondary prevention will never experience adequate ICD therapy during the 24–60 months of follow-up after device implantation [4,5,6,7].

The prevalence of frailty syndrome in European society (65 years and older) ranges from 5.8% to 27.3%. Between 34.6% and 50.9% of older adult populations are considered to be "prefrail" [8]. Frailty syndrome is a complex multidimensional state that is associated with a reduction in physiological reserves and resistance to stress factors due to the reduced efficiency of organs and systems [9]. It is estimated that frailty syndrome is diagnosed three times more often in people with cardiovascular diseases than in the population without cardiovascular diseases, which results in higher mortality and subsequent hospitalizations. The diagnosis of frailty syndrome in the elderly population is considered to be an essential element for correct therapeutic decision-making and cardiovascular risk stratification [10,11,12].

Studies have shown that the need to consider a frailty assessment when making decisions about treatment is critical. Treating elderly patients is often associated with a high risk of complications. The incorporation of a frailty analysis into the risk stratification can help to differentiate older patients who could benefit from an intervention from those for whom aggressive intervention might cause a deterioration in their health [13,14]. Patients with ventricular arrhythmias that have been treated with an implantable ICD constitute a specific subgroup of patients. Conducting research to assess the prevalence of the frailty syndrome in this particular patient population is an important factor that may affect the fate of a patient. 

The aim of the study was to identify the prevalence of frailty syndrome in elderly patients with an ICD and to assess the impact of the diagnosis of frailty syndrome on the future outcomes of those patients.

## 2. Materials and Methods

### 2.1. Study Design and Settings 

A cross-sectional study was performed to achieve the study objectives. Based on the population size, fraction size, and maximum error with a 95% confidence level, the minimum number of participants in the sample was calculated to be 93 patients. The data to calculate the minimum number of participants in the group was obtained from the European Heart Rhythm Association (EHRA) White Book 2017 [15].

### 2.2. Study Participants and Selection 

A total of 103 consecutive patients 60 years old or older were included in the study. The patients (85 men and 18 women, average age 71.56–8.17 years) were hospitalized in the Department of Electrocardiology and Heart Failure, Katowice, Poland. All of the patients included in the study had an implanted single or dual-chamber ICD. In the research, there was a 12-month follow-up. The criteria for inclusion into the study were as follows: age more than 60 years, indications for the implantation of a cardioverter-defibrillator, and consent to participate in the study and the follow-up visit. Patients with indications for cardiac resynchronization therapy (CRT) with an implanted ICD (CRT-D), with an active neoplastic disease in the active phase, mental illness, or an incomplete questionnaire were excluded from the study. All of the patients were optimally pharmacologically treated for cardiovascular causes and had a physical exam and echocardiographic and 12-lead electrocardiograms performed before ICD implantation. During the observation, the presence of ICD electric shocks, inadequate electric shocks, and the presence of an electric storm, which was defined as 3 or more ventricular tachyarrhythmias requiring electrotherapy within 24 h, were analyzed. The event data came from the ICD external analyzers.

### 2.3. Ethical Considerations

The study protocol was approved by the Bioethical Committee of the Medical University of Silesia, number (KNW/0022/KB/36/18). The study protocol was carried out in accordance with the Helsinki Convention. The participation of patients in the study was completely anonymous and voluntary; all participants gave their voluntary consent to participate in the study and were also informed about its purpose. Patients had the option to withdraw from the study at any stage.

### 2.4. Research Instruments

The occurrence of frailty syndrome was assessed in all of the patients that were included in the study using the Tilburg Frailty Indicator scale (TFI) on the day of their admission to the hospital. The Tilburg Frailty Indicator is a tool that is used to assess the occurrence of frailty developed by Gobbens et al. in 2010 [16]. The scale is divided into two parts: part A, sociodemographic characteristics, and part B, which contains 15 questions about frailty syndrome. The tool enables frailty syndrome to be assessed in three domains: physical, psychological, and social. There are eight components for the physical domain, four components for the psychological domain, and three components for the social domain. It is possible to obtain a minimum of 0 points and a maximum of 15 points on the scale. Frailty syndrome is recognized as a score of five points or more. The greater the number of points that are obtained, the higher the frailty syndrome level [17].

### 2.5. Statistical Analysis

The data obtained in the study were analyzed in order to check the normality of the distribution using the Shapiro–Wilk test. The Fisher Exact test or chi-square test with Yates’s correction was also used for selected non-parametric data where possible. The Kruskal–Wallis test was used to compare non-parametric data. Kaplan–Meier curves were used to assess the survival and event-free periods (electric shock, inadequate electric shocks, electrical storms). Multivariate logistic regression analysis was used to predict the factors associated with the ICD functioning with additional corrections for age, gender, and body mass index. The ROC analysis was used to evaluate the diagnostic usefulness of frailty syndrome. The analyses were performed using MedCalc software (MedCalc Software Ltd., 8400 Ostend, Belgium).

## 3. Results

All of the patients had a follow-up visit within 365 ± 12 days of the implantation of a cardioverter-defibrillator. Adequate ICD electric shock occurred in 28 (27.2%), while inadequate electric shock occurred in 11 (10.7%) of the patients. Only three (2.9%) percent experienced an electrical storm. In nine (8.7%) patients, both adequate and inadequate electric shocks were observed. The characteristics of the patients included in the study, including the endpoints, are presented in Table 1.

Most of the patients (60.2%) had ischemic etiology. The mean left ventricular ejection fraction was 28.1% ± 5.4, the mean width of the QRS complexes was 100.2 ms ± 13.4, and the mean heart rate was 71.0 ± 21.6. In our population, the sinus rhythm was dominant (77.7%). In the remaining patients, atrial fibrillation was diagnosed as the leading rhythm. Of all patients, 38.4% were in NYHA class 1, 31.1% in class 2; in the rest, no symptoms of heart failure were found.

All enrolled in the study had optimized pharmacotherapy prior to implantation of ICD, in particular, B-blocker (100% included), angiotensin-converting enzyme inhibitors, ACE (66.9%); mineralocorticoid receptor antagonists, MRA (51.5%); and other diuretics (49.5%). The most common disease was arterial hypertension (75.7%), diabetes was diagnosed in 42.7%, renal failure in 8.7%, and chronic obstructive pulmonary disease, COPD in 5.8%.

Frailty syndrome was diagnosed in 78 (75.73%) of the patients that were included in the study. The mean values of the TFI were 6.55 ± 2.67, in the physical domain 4.06 ± 1.79, in the psychological domain 2.06 ± 1.10, and in the social domain 0.44 ± 0.55. The details are presented in Table 2.

### Endpoint Analysis

During the follow-up period, 28 (27.2%) patients had a defibrillator cardioverter electric shock, which occurred statistically more often in patients with diagnosed frailty syndrome; 27 (34.6%), compared to the robust patients, one (4%); *p* = 0.0148. A similar observation was made when the occurrence of inadequate electric shocks was compared in the patients with frailty syndrome and the robust patients. Inadequate electric shocks occurred more often in patients with frailty syndrome; 11 (14.1%), compared to the robust patients for whom there were no inadequate shocks. A similar observation was made for the differences in the occurrence of an electrical storm when the patients with frailty syndrome; 3 (3.84%), and robust (0%), were compared. 

In the multivariate logistic regression with additional corrections for age, gender, body mass index, frailty (OR: 1,203, 95% CI:1.0126–1.4298; *p* < 0.030) was an independent predictor of a defibrillator cardioverter electric shock. Similarly, in the multivariate logistic regression with additional corrections for age, gender, body mass index, frailty (OR: 1.3623, 95% CI:1.0290–1.8035; *p* = 0.019) was also an independent predictor for inadequate electric shocks. In the multivariate logistic regression with additional corrections for age, gender, body mass index, there was no evidence that frailty syndrome is an independent factor in the occurrence of an electrical storm (OR: 1.2537, 95% CI:0.7760–2.0371; *p* = 0.238). No other factors proved to be predictors of endpoints. 

A multivariate regression model was performed. The predictors were heart failure baseline characteristics, medication, and the presence of frailty syndrome. The dependent variable was a defibrillator cardioverter electric shock. The model was statistically significant and explained 14% of the observed variance in the dependent variable (*p* = 0.0006, R2 = 0.1373). The analysis showed that frailty (*p* = 0.0039) and the baseline rhythm (*p* = 0.0039) are important predictors of the dependent variable.

**Figure 1:**
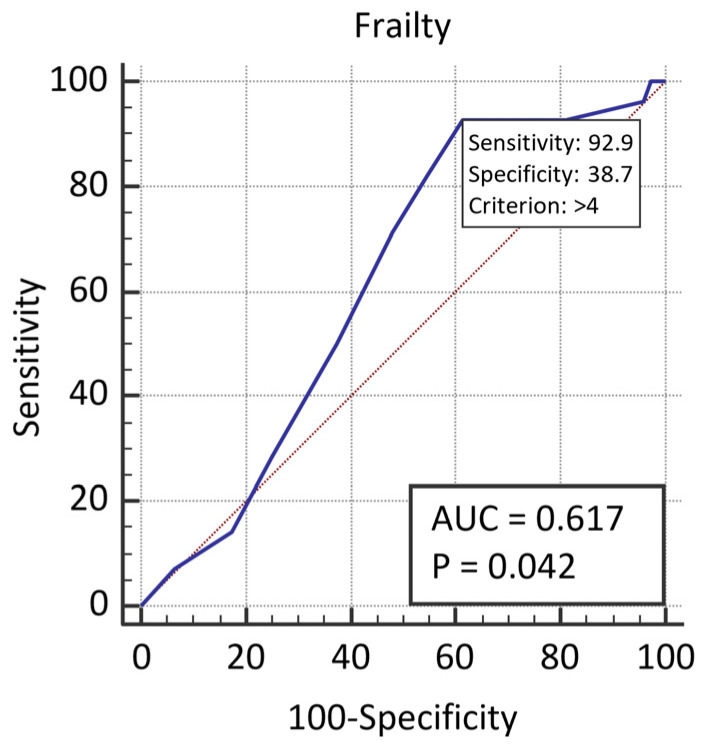
The ROC curves for the prediction of an electric shock of an ICD by frailty.

Figure 1 presents the ROC curve for the prediction of an electric shock of an ICD by frailty. The area under the curve was 0.617(95% CI = 0.516–0.711), the cut-off value for a designation of frailty was >4 (*p* = 0.0420). 

**Figure 2:**
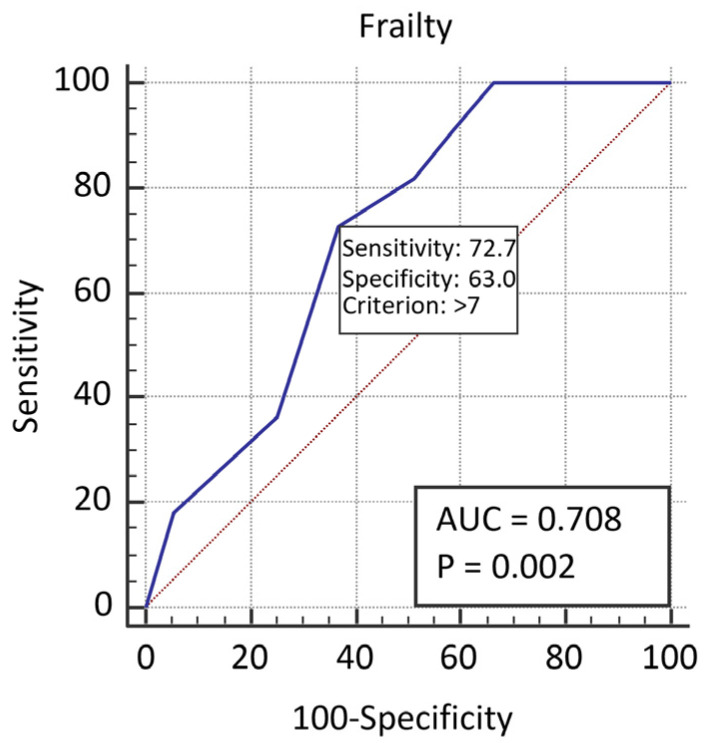
The ROC curves for the prediction of occurrence inappropriate shocks by frailty.

In the ROC curve, which is graphically presented in Figure 2, for the prediction of occurrence, inappropriate shocks by frailty, the area under the curve was 0.708(95% CI = 0.610–0.793). The cut-off value for the recognition of frailty was > 7 (*p* = 0.0021). 

Both areas demonstrate that frailty alone is not a very good predictor of shocks.

In the ROC curves for frailty for the occurrence of an electrical storm, the area under the curve was 0.708(95% CI=0.562–0.752). The cut-off value for the frailty recognition was >4; however, the values were not statistically significant (*p* = 0.376). 

**Figure 3:**
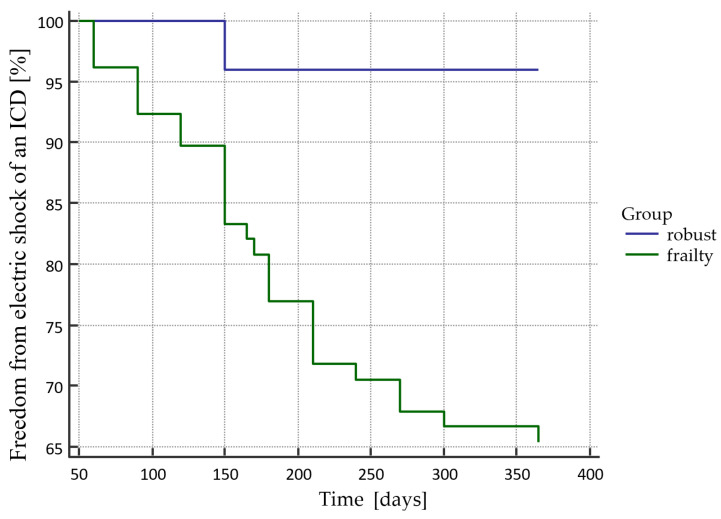
Freedom from an electric shock of an ICD in the Kaplan–Mayer curves according to the presence of frailty.

**Figure 4:**
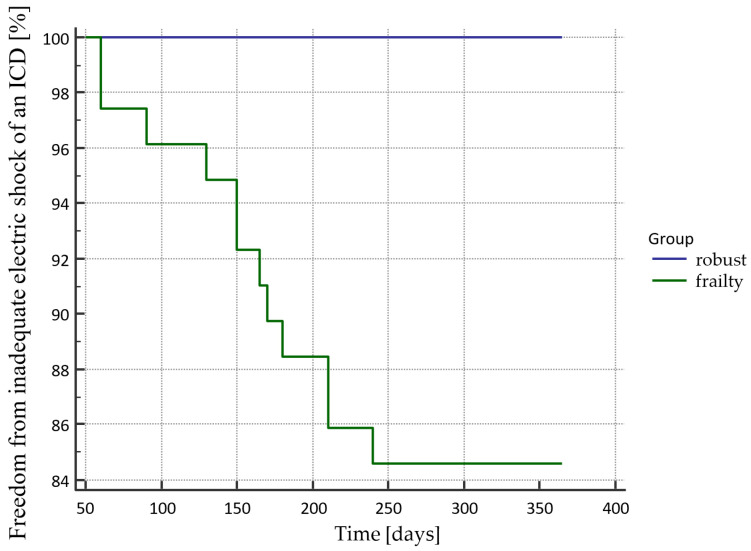
Freedom from an inadequate electric shock of an ICD in the Kaplan–Mayer curves according to the presence of frailty.

Freedom from an electric shock of an ICD according to the presence of frailty is presented in Figure 3; the difference was statistically significant (*p* = 0.0044). An analogous situation occurred in the case of inadequate electric shocks, and the differences were statistically significant (*p* = 0.0414). The curve is presented in Figure 4. The analysis of the electric storm episodes showed that these events occurred in patients with recognized frailty syndrome but did not occur in patients without frailty syndrome.

## 4. Discussion

ICDs are widely used in elderly patients. The AVID and MADIT-II studies, which analyzed subgroups, showed equivalent benefits of ICD implantation in both older and younger patients [18,19]. The meta-analysis of Santangeli et al. showed that treatment by implanting an ICD might be less beneficial for elderly patients who have a severe left ventricle dysfunction (HR 0.75; 95% CI 0.61–0.91) [20]. Elderly patients had a differentiation in the frequency of electric shocks and inadequate electric shocks that was dependent on the occurrence of frailty syndrome. There was no such differentiation in the frequency of electrical storms. The combined data from clinical trials revealed that treatment using an ICD significantly reduces the overall mortality and the incidence of arrhythmic deaths in patients ≤75 years old, but not for patients ≥75 years old [21]. Observational studies and data from numerous registers have proven that age should not be a factor that excludes ICD implantation [2,22,23]. 

Frailty is a relatively new syndrome that has been recognized in the geriatric population. In recent years, many scientific reports have shown its role in the prediction of a disease diagnosis and also in the treatment of elderly patients. The interaction between frailty syndrome and ICD efficacy has not yet been defined because frailty syndrome has not been a factor that has been analyzed in large clinical trials. It was found that patients with diagnosed frailty syndrome have a 22% risk of mortality after one year compared to 12% of robust patients [24]. In our study, frailty syndrome occurred in 75% percent of the patients and was an independent factor for electric shocks and inadequate electric shocks. 27.2% of our patients experienced a defibrillator cardioverter electric shock. Shocks occurred in 34.6% compared to the robust patients (4%). Similar results were found in the occurrence of inadequate electric shocks (14.1% vs. 0%). These results confirm the necessity of a frailty evaluation before ICD implantation. A small correction of cut-off values can improve the usefulness of frailty recognition. 

In the paper by Mlynarska et al., which concerned patients with an implanted CRT, it was shown that the occurrence of frailty syndrome does not affect the incidence of electrical storms but that it is a factor for more frequent decompensations and rehospitalizations. Similar results were obtained in this study in which frailty syndrome was not a risk factor for the frequent occurrence of an electrical storm [25].

Some studies have shown a similar percentage of the complications that are associated with ICD implantation between different age groups, as well as a higher risk of inadequate shock in younger patients [26,27,28]. In the presented study, only two lead dislocations occurred. In both cases, the issue was resolved by repositioning them. There was one small device pocket problem, but it was possible to treat it conservatively. These numbers were too small to be statistically analyzed. The frequent and inadequate electric shocks in the older age group could be explained by the presence of frailty syndrome. When frailty syndrome is diagnosed, age is not a factor in the occurrence of complications. Two large observational studies showed that age is not an independent risk factor for the increased complications that are associated with lead dislocation, lead damage, or infection [29,30,31].

In a study by Bardy et al., the authors showed that the rationale for ICD therapy is independent of age and is comparable in older and younger subjects. Older patients do not appear to be at greater risk of inappropriate electric shocks [32]. The results of the MADIT-RIT study indicated a potency in patients with inadequate or unnecessary electric shocks [33]. 

Frailty syndrome is also associated with increased concerns about the ability to function with an implanted ICD. The reason for the increased intensity of the fears may be the increased number of electric shocks or inadequate interventions. Hence, patients with frailty syndrome who have an increased number of device interventions have more concerns about the perceived limitations and device-specific concerns [34].

## 5. Conclusions

About three-quarters of elderly patients who had qualified for ICD implantation had frailty syndrome. Adequate and inadequate electric shocks occurred more often in the frailty subgroup compared to the robust patients. Frailty seems to be an independent predictor of a defibrillator cardioverter electric shock as well as a predictor for an inadequate electric shock. 

## Figures and Tables

**Table 1:** The characteristics of the elderly patients included in the study, including the endpoints.

	Overall Population n=103 Median; [Q1;Q3]	With No Shock n=75 Median; [Q1;Q3]	Electric Shock n=28 Median; [Q1;Q3]	Inadequate Electric Shock n=11 Median; [Q1;Q3]	Electrical Storm n=3 Median; [Q1;Q3]	*p*
Age [years]	71; [64;78]	71; [64;78]	70.5; [65;79]	67; [64;73]	74; [63;85]	0.8335 ^#^
Gender woman	17.47%	21.33%	7.14%	18.18%	66.67%	0.0625 *
Weight [kg]	79; [70;88]	80.3; [67;91]	77; [72;83]	72.5; [71;75]	69; [66;72]	0.3260 ^#^
BMI	28.44; [25.28;31.63]	28.51; [24.86;31.6]	26.64; [24.96;30.76]	26.16; [24.93;27.42]	29.47; [28.19;30.76]	0.5804 ^#^
Place of residence - urban area - rural area	81.55% 18.45%	85.34% 14.66%	67.86% 32.14%	45.45% 54.55%	33.33% 66.67%	0.0043 *
Education - none or primary - secondary - vocational or higher	37.87% 61.16% 0.97%	36.00% 62.67% 1.33%	42.85% 57.14% 0	36.37% 63.63% 0	66.67% 33.33% 0	0.6941*
Marital Status - married/living with a partner - unmarried - widow/widower	90.29% 7.77% 1.94%	93.33% 4.00% 2.66%	85.71% 14.29% 0	100% 0 0	100% 0 0	0.2231 *
Professional status Working Retired Pensioner	21.36% 71.84% 6.80%	22.66% 72.00% 5.34%	17.85% 75.00% 7.14%	18.19% 9.09% 72.72%	0 66.67% 33.33%	0.8419 *
Smoking	29.13%	32.00%	21.42%	18.18%	33.33%	0.6252 *
Indication for implantation − primary	49.51%	53.33%	39.28%	27.27%	33.33%	0.2816 *
More than two diseases	77.67%	77.33%	86.57%	81.81%	100%	0.0001 *

Abbreviation: BMI—Body Mass Index; Q1—first quartile; Q3—third quartile. * chi-squared test with Yates’s correction; ^#^ Kruskal–Wallis test.

**Table 2:** Number of points obtained in the total TFI score as well as in its domains according to the electric shocks.

TFI and Domain	Overall Population	With No Shock	Electric Shock	Inadequate Electric Shock	Electrical Storm	*p*
General (mean ± SD Median; [Q1;Q3])	6.66 ± 2.66 7; [4;9]	6.27 ± 2.76 6; [4;8]	7.38 ± 2.21 7.5; [6;9]	8.56 ± 1.94 8; [5;11]	8.00 ± 3.00 8; [5;11]	0.0436 ^#^
Physical (mean ± SD Median; [Q1;Q3])	4.58 ± 1.79 4; [3;6]	3.83 ± 1.82 4; [2;5]	4.73 ± 1.56 5; [4;6]	5.22 ± 1.30 5; [3;6]	4.67 ± 1.53 5; [3;6]	0.0444 ^#^
Psychological (mean ± SD Median; [Q1;Q3])	2.06 ± 1.11 2; [1;3]	2.04 ± 1.14 2; [1;3]	2.11 ± 0.99 2; [1;3]	2.67 ± 0.87 3; [2;3]	2.67 ± 0.58 3; [2;3]	0.2856 ^#^
Social (mean ± SD Median; [Q1;Q3])	0.44 ± 0.55 0; [0;1]	0.40 ± 0.54 0; [0;1]	0.54 ± 0.58 0.5; [0;1]	0.67 ± 0.71 0; [0;2]	0.67 ± 1.15 0; [0;2]	0.6767 ^#^

Abbreviation: Q1—first quartile; Q3—third quartile; SD—standard deviation; TFI—Tilburg Frailty Indicator. ^#^ Kruskal–Wallis test.

## Data Availability

The datasets used and/or analyses during the current study are available from the corresponding author on reasonable request.
